# (*R*)-1-(4-Bromo­benzo­yl)-4-(1-phenyl­prop­yl)thio­semicarbazide

**DOI:** 10.1107/S1600536808006806

**Published:** 2008-04-10

**Authors:** Monazza Serwer, M. Khawar Rauf, Masahiro Ebihara, Shahid Hameed

**Affiliations:** a Department of Chemistry, Quaid-i-Azam University, Islamabad 45320, Pakistan; b Department of Chemistry, Faculty of Engineering, Gifu University, Yanagido, Gifu 501-1193, Japan

## Abstract

The title compound, C_17_H_18_BrN_3_OS, is an important inter­mediate for the synthesis of biologically active heterocyclic compounds. The thio­urea group is approximately planar. The crystal structure is stabilized by inter­molecular N—H⋯O hydrogen-bonding inter­actions.

## Related literature

For related literature, see: Akhtar *et al.* (2006 ▶, 2007 ▶); Cardia *et al.* (2006 ▶); Dolman *et al.* (2006 ▶); Hassan *et al.* (2006 ▶); Jalilian *et al.* (2000 ▶); Kucukguzel *et al.* (2006 ▶); Mohareb *et al.* (2007 ▶); Singh *et al.* (2003 ▶, 2005 ▶).
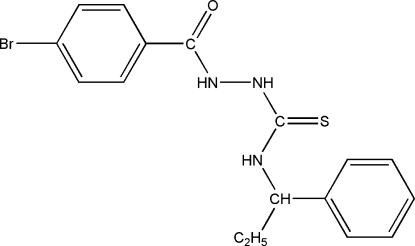


## Experimental

### 

#### Crystal data

C_17_H_18_BrN_3_OS
*M* *_r_* = 392.31
Orthorhombic, 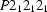

*a* = 6.263 (3) Å
*b* = 9.698 (5) Å
*c* = 27.651 (15) Å
*V* = 1679.5 (15) Å^3^
*Z* = 4
Mo *K*α radiation
μ = 2.58 mm^−1^
*T* = 123 (2) K
0.30 × 0.25 × 0.20 mm

#### Data collection

Rigaku/MSC Mercury CCD diffractometer
Absorption correction: integration (*NUMABS*; Higashi, 1999 ▶) *T*_min_ = 0.512, *T*_max_ = 0.626
13732 measured reflections
3824 independent reflections
3516 reflections with *I* > 2σ(*I*)
*R*_int_ = 0.057

#### Refinement

*R*[*F* ^2^ > 2σ(*F* ^2^)] = 0.048
*wR*(*F* ^2^) = 0.074
*S* = 1.13
3824 reflections
219 parameters
H atoms treated by a mixture of independent and constrained refinement
Δρ_max_ = 0.34 e Å^−3^
Δρ_min_ = −0.35 e Å^−3^
Absolute structure: Flack (1983 ▶), 1584 Friedel pairs
Flack parameter: 0.020 (10)

### 

Data collection: *CrystalClear* (Molecular Structure Corporation & Rigaku, 2001 ▶); cell refinement: *CrystalClear*; data reduction: *TEXSAN* (Molecular Structure Corporation & Rigaku, 2004 ▶); program(s) used to solve structure: *SIR97* (Altomare *et al.*, 1999 ▶); program(s) used to refine structure: *SHELXL97* (Sheldrick, 2008 ▶); molecular graphics: *ORTEPII* (Johnson, 1976 ▶); software used to prepare material for publication: *SHELXL97* and *TEXSAN*.

## Supplementary Material

Crystal structure: contains datablocks I, global. DOI: 10.1107/S1600536808006806/hg2385sup1.cif

Structure factors: contains datablocks I. DOI: 10.1107/S1600536808006806/hg2385Isup2.hkl

Additional supplementary materials:  crystallographic information; 3D view; checkCIF report

## Figures and Tables

**Table 1:** Hydrogen-bond geometry (Å, °)

*D*—H⋯*A*	*D*—H	H⋯*A*	*D*⋯*A*	*D*—H⋯*A*
N1—H1⋯O1^i^	0.84 (4)	2.03 (4)	2.834 (4)	161 (4)

Symmetry code: (i) 
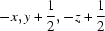
.

**Fig. 1.**
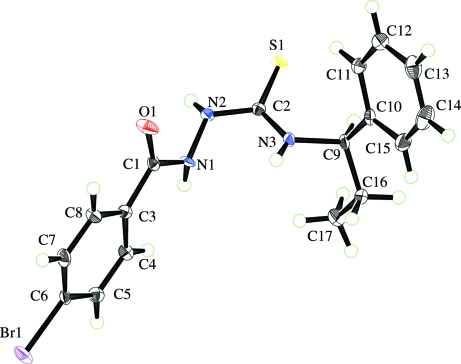
Molecular structure of (I) showing atom-labelling scheme and displacement ellipsoids at the 30% probability level.

**Fig. 2.**
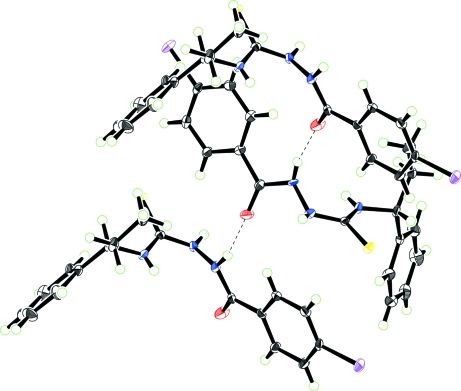
Showing hydrogen bonded molecules through N—H···O.

## supplementary crystallographic information

### Comment

Thiosemicarbazides have attracted much attention partly because of their biological activities such as antifungal (Mohareb *et al.*, 2007), antibacterial (Kucukguzel *et al.*, 2006), antiamoebic (Singh *et al.*, 2005) antitubercular (Cardia *et al.*,2006), corrosion inhibitors (Singh *et al.*, 2003) and partly because of their use as intermediates in the synthesis of many biologically active heterocyclic compounds like 1,3,4-oxadiazoles (Dolman *et al.*, 2006), 1,3,4-thiadiazoles (Jalilian *et al.*,2000) and 1,2,4-triazole (Akhtar *et al.*, 2007; Akhtar *et al.*, 2006). The other important biologically active compounds synthesized from thiosemicarbazides include thiazoles, thiazines, thiadiazines, pyrazines and indazoles (Hassan *et al.*, 2006). The C2—S1 and C1—O1 bonds both show the expected full double-bond character, while the short values for the C1—N1, C2—N2, N1—N2,C2—N3 and C9—N3 bond lengths also indicate partial double-bond character. The thiourea group is approximately planar. The crystal packing is stabilized by N(1)—H(1)···O(1) and N(3)—H(3)···S(1) hydrogen bonds.

### Experimental

The 4-bromobenzoic acid hydrazide (0.0068 moles) was dissolved in methanol (30ml) and a solution of 0.0066 moles of R-(+)-1-phenylpropylisothiocyanate, separately dissolved in 10 ml of methanol, was added drop wise with continuous stirring. The reaction mixture was refluxed and after consumption of the starting materials (tlc), the mixture was cooled to room temperature and methanol evaporated in vacuo. The crude thiosemicarbazide was recrystallized from a mixture of ethyl acetate and petroleum ether. Yield: 85%;  m.p 160-161 °C; R_f_: 0.34 (Petroleum ether: acetone; 6:4); IR (ν_max_, KBr, cm^-1^): 3378, 3273, 3191, 3033, 2967, 2877, 1669, 1238, 1591, 1528, 699; ^1^H-NMR (Acetone-d_6_): δ 9.81 (1H, s), 8.58 (1H, s), 8.06 (1H, s), 0.97 (3H, t, J = 7.5 Hz), 1.92-1.82 (2H, m), 5.58 (1H, dd, J = 15.6, 7.2 Hz), 7.39 (2H, dd, J = 7.2, 1.5 Hz ), 7.30 (2H, dt, J = 7.5, 3.0 Hz), 7.22 (1H, dt, J = 7.2, 3.0 Hz), 7.92 (2H, d, J = 8.4 Hz), 7.72 (2H, d, J = 8.7 Hz); ^13^C-NMR (Acetone-d_6_):δ 183.51, 165.60, 142.88, 133.20, 131.64, 129.61, 128.10, 126.87, 126.74,126.12, 59.55, 28.21, 10.47; EIMS: (m/z %) 214 (20), 183 (100), 155 (55),104 (10), 76 (50), 50 (45). Elemental analysis for C_17_H_18_N_3_SOBr (391):C, 52.05; H, 4.62; N, 10.71; S, 8.17. Found: C, 51.96; H, 4.70; N, 10.82; S, 8.07.

### Refinement

H atom on the N atom was refined isotropically. Other H atoms were placed in idealized positions and treated as riding atoms with C—H distance in the range 0.95–0.99 Å and *U*_iso_(H) = 1.2*U*_eq_(C) or 1.5*U*_eq_(C).

### Crystal data

**Table:**

C_17_H_18_BrN_3_OS	*F*_000_ = 800
*M**_r_* = 392.31	*D*_x_ = 1.552 Mg m^−^^3^
Orthorhombic, *P*2_1_2_1_2_1_	Mo *K*α radiation λ = 0.71070 Å
Hall symbol: P 2ac 2ab	Cell parameters from 4820 reflections
*a* = 6.263 (3) Å	θ = 3.1–27.5º
*b* = 9.698 (5) Å	µ = 2.58 mm^−^^1^
*c* = 27.651 (15) Å	*T* = 123 (2) K
*V* = 1679.5 (15) Å^3^	Block, colourles
*Z* = 4	0.30 × 0.25 × 0.20 mm

### Data collection

**Table:**

Rigaku/MSC Mercury CCD diffractometer	3824 independent reflections
Radiation source: fine-focus sealed tube	3516 reflections with *I* > 2σ(*I*)
Monochromator: graphite	*R*_int_ = 0.057
*T* = 123(2) K	θ_max_ = 27.5º
ω scans	θ_min_ = 3.1º
Absorption correction: integration(NUMABS; Higashi, 1999)	*h* = −8→8
*T*_min_ = 0.512, *T*_max_ = 0.627	*k* = −12→8
13732 measured reflections	*l* = −26→35

### Refinement

**Table:**

Refinement on *F*^2^	H atoms treated by a mixture of independent and constrained refinement
Least-squares matrix: full	*w* = 1/[σ^2^(*F*_o_^2^) + 1.3691*P*] where *P* = (*F*_o_^2^ + 2*F*_c_^2^)/3
*R*[*F*^2^ > 2σ(*F*^2^)] = 0.048	(Δ/σ)_max_ < 0.001
*wR*(*F*^2^) = 0.074	Δρ_max_ = 0.34 e Å^−^^3^
*S* = 1.13	Δρ_min_ = −0.35 e Å^−^^3^
3824 reflections	Extinction correction: SHELXL97 (Sheldrick, 2008), Fc^*^=kFc[1+0.001xFc^2^λ^3^/sin(2θ)]^-1/4^
219 parameters	Extinction coefficient: 0.0018 (4)
Primary atom site location: structure-invariant direct methods	Absolute structure: Flack (1983), 1584 Friedel pairs
Secondary atom site location: difference Fourier map	Flack parameter: 0.020 (10)
Hydrogen site location: inferred from neighbouring sites	

### Special details

**Table:**

Geometry. All e.s.d.'s (except the e.s.d. in the dihedral angle between two l.s. planes) are estimated using the full covariance matrix. The cell e.s.d.'s are taken into account individually in the estimation of e.s.d.'s in distances, angles and torsion angles; correlations between e.s.d.'s in cell parameters are only used when they are defined by crystal symmetry. An approximate (isotropic) treatment of cell e.s.d.'s is used for estimating e.s.d.'s involving l.s. planes.
Refinement. Refinement of *F*^2^ against ALL reflections. The weighted *R*-factor *wR* and goodness of fit *S* are based on *F*^2^, conventional *R*-factors *R* are based on *F*, with *F* set to zero for negative *F*^2^. The threshold expression of *F*^2^ > σ(*F*^2^) is used only for calculating *R*-factors(gt) *etc*. and is not relevant to the choice of reflections for refinement. *R*-factors based on *F*^2^ are statistically about twice as large as those based on *F*, and *R*- factors based on ALL data will be even larger.

### Fractional atomic coordinates and isotropic or equivalent isotropic displacement parameters (Å^2^)

**Table:**

	*x*	*y*	*z*	*U*_iso_*/*U*_eq_	
C1	0.0742 (6)	0.2098 (3)	0.24009 (12)	0.0141 (8)	
O1	0.0089 (4)	0.0922 (2)	0.23367 (10)	0.0260 (7)	
N1	−0.0306 (5)	0.3005 (3)	0.26956 (12)	0.0144 (7)	
H1	0.006 (6)	0.384 (4)	0.2666 (13)	0.017*	
C2	−0.2924 (6)	0.2804 (3)	0.33209 (13)	0.0124 (8)	
S1	−0.55059 (15)	0.30911 (9)	0.34643 (4)	0.0169 (2)	
N2	−0.2383 (5)	0.2701 (3)	0.28467 (12)	0.0144 (7)	
H2	−0.319 (7)	0.288 (4)	0.2668 (15)	0.017*	
N3	−0.1369 (5)	0.2639 (3)	0.36436 (12)	0.0146 (6)	
H3	−0.024 (6)	0.254 (3)	0.3547 (15)	0.017*	
C3	0.2725 (6)	0.2613 (3)	0.21649 (13)	0.0134 (8)	
C4	0.3408 (6)	0.3985 (4)	0.21787 (13)	0.0156 (8)	
H4	0.2625	0.4643	0.2361	0.019*	
C5	0.5216 (6)	0.4390 (3)	0.19285 (14)	0.0171 (8)	
H5	0.5681	0.5322	0.1939	0.020*	
C6	0.6334 (7)	0.3426 (3)	0.16636 (12)	0.0153 (7)	
C7	0.5710 (6)	0.2056 (4)	0.16486 (13)	0.0197 (8)	
H7	0.6508	0.1401	0.1468	0.024*	
C8	0.3915 (7)	0.1659 (3)	0.18998 (13)	0.0169 (8)	
H8	0.3479	0.0721	0.1893	0.020*	
Br1	0.87772 (7)	0.39971 (4)	0.131114 (15)	0.02482 (12)	
C9	−0.1593 (6)	0.2802 (3)	0.41665 (12)	0.0152 (8)	
H9	−0.3033	0.3217	0.4224	0.018*	
C10	−0.1581 (6)	0.1398 (3)	0.44199 (13)	0.0154 (8)	
C11	−0.3136 (6)	0.0440 (3)	0.42905 (14)	0.0207 (9)	
H11	−0.4171	0.0674	0.4053	0.025*	
C12	−0.3188 (7)	−0.0852 (4)	0.45040 (15)	0.0258 (10)	
H12	−0.4244	−0.1503	0.4410	0.031*	
C13	−0.1711 (7)	−0.1193 (4)	0.48521 (14)	0.0273 (10)	
H13	−0.1748	−0.2078	0.4999	0.033*	
C14	−0.0166 (7)	−0.0245 (4)	0.49888 (16)	0.0305 (11)	
H14	0.0848	−0.0475	0.5231	0.037*	
C15	−0.0110 (6)	0.1039 (4)	0.47705 (14)	0.0231 (9)	
H15	0.0956	0.1683	0.4863	0.028*	
C16	0.0052 (6)	0.3842 (4)	0.43521 (13)	0.0200 (8)	
H16A	0.1506	0.3475	0.4296	0.024*	
H16B	−0.0137	0.3966	0.4705	0.024*	
C17	−0.0165 (7)	0.5243 (4)	0.41003 (15)	0.0276 (10)	
H17A	0.0209	0.5147	0.3758	0.041*	
H17B	0.0798	0.5909	0.4254	0.041*	
H17C	−0.1641	0.5569	0.4129	0.041*	

### Atomic displacement parameters (Å^2^)

**Table:**

	*U*^11^	*U*^22^	*U*^33^	*U*^12^	*U*^13^	*U*^23^
C1	0.017 (2)	0.0147 (16)	0.0109 (19)	0.0004 (14)	0.0000 (16)	0.0045 (13)
O1	0.0226 (16)	0.0156 (12)	0.0397 (18)	−0.0052 (12)	0.0131 (13)	−0.0043 (12)
N1	0.0114 (16)	0.0133 (13)	0.0185 (19)	−0.0054 (12)	0.0049 (14)	0.0010 (12)
C2	0.0131 (19)	0.0096 (15)	0.015 (2)	−0.0006 (13)	0.0013 (16)	−0.0013 (13)
S1	0.0102 (5)	0.0220 (4)	0.0184 (5)	0.0014 (4)	0.0029 (4)	0.0007 (4)
N2	0.0078 (17)	0.0215 (16)	0.0140 (19)	−0.0001 (13)	−0.0027 (14)	0.0057 (13)
N3	0.0102 (15)	0.0230 (13)	0.0105 (16)	0.0013 (12)	0.0013 (17)	−0.0019 (12)
C3	0.0120 (19)	0.0140 (16)	0.014 (2)	0.0009 (13)	−0.0015 (16)	0.0044 (13)
C4	0.015 (2)	0.0156 (15)	0.0165 (19)	0.0032 (17)	0.0005 (16)	0.0038 (15)
C5	0.018 (2)	0.0155 (17)	0.018 (2)	−0.0002 (14)	−0.0004 (18)	0.0023 (14)
C6	0.0116 (18)	0.0256 (16)	0.0087 (18)	0.0002 (15)	0.0002 (18)	0.0040 (13)
C7	0.020 (2)	0.0242 (17)	0.015 (2)	0.0037 (15)	0.0044 (17)	−0.0027 (15)
C8	0.016 (2)	0.0158 (15)	0.019 (2)	−0.0009 (15)	0.0043 (19)	0.0004 (13)
Br1	0.01688 (19)	0.03197 (19)	0.0256 (2)	−0.00092 (17)	0.0097 (2)	0.00672 (18)
C9	0.015 (2)	0.0230 (17)	0.0078 (19)	−0.0003 (15)	0.0031 (16)	−0.0007 (13)
C10	0.018 (2)	0.0194 (16)	0.0090 (18)	0.0018 (14)	0.0014 (17)	−0.0006 (12)
C11	0.020 (2)	0.0270 (18)	0.015 (2)	−0.0036 (15)	−0.0038 (17)	0.0045 (15)
C12	0.028 (3)	0.028 (2)	0.021 (2)	−0.0071 (18)	0.0013 (18)	−0.0019 (17)
C13	0.038 (3)	0.0230 (19)	0.021 (2)	0.0071 (18)	0.005 (2)	0.0009 (16)
C14	0.035 (3)	0.033 (2)	0.023 (3)	0.0116 (19)	−0.011 (2)	0.0005 (18)
C15	0.027 (2)	0.0242 (18)	0.018 (2)	−0.0021 (19)	−0.0045 (18)	0.0018 (18)
C16	0.020 (2)	0.0275 (18)	0.013 (2)	−0.0051 (16)	0.0007 (16)	−0.0030 (16)
C17	0.032 (3)	0.030 (2)	0.021 (2)	−0.0109 (18)	0.004 (2)	0.0027 (17)

### Geometric parameters (Å, °)

**Table:**

C1—O1	1.224 (4)	C8—H8	0.9500
C1—N1	1.367 (4)	C9—C16	1.531 (5)
C1—C3	1.489 (5)	C9—C10	1.532 (4)
N1—N2	1.397 (4)	C9—H9	1.0000
N1—H1	0.84 (4)	C10—C15	1.382 (5)
C2—N3	1.330 (5)	C10—C11	1.393 (5)
C2—N2	1.358 (5)	C11—C12	1.386 (5)
C2—S1	1.688 (4)	C11—H11	0.9500
N2—H2	0.73 (4)	C12—C13	1.376 (6)
N3—C9	1.461 (5)	C12—H12	0.9500
N3—H3	0.76 (4)	C13—C14	1.387 (6)
C3—C8	1.396 (5)	C13—H13	0.9500
C3—C4	1.398 (5)	C14—C15	1.384 (5)
C4—C5	1.384 (5)	C14—H14	0.9500
C4—H4	0.9500	C15—H15	0.9500
C5—C6	1.379 (5)	C16—C17	1.532 (5)
C5—H5	0.9500	C16—H16A	0.9900
C6—C7	1.386 (5)	C16—H16B	0.9900
C6—Br1	1.897 (4)	C17—H17A	0.9800
C7—C8	1.376 (5)	C17—H17B	0.9800
C7—H7	0.9500	C17—H17C	0.9800
			
O1—C1—N1	121.7 (3)	C16—C9—C10	115.4 (3)
O1—C1—C3	121.8 (3)	N3—C9—H9	106.8
N1—C1—C3	116.5 (3)	C16—C9—H9	106.8
C1—N1—N2	119.3 (3)	C10—C9—H9	106.8
C1—N1—H1	115 (3)	C15—C10—C11	118.6 (3)
N2—N1—H1	119 (3)	C15—C10—C9	123.3 (3)
N3—C2—N2	117.1 (3)	C11—C10—C9	118.2 (3)
N3—C2—S1	124.3 (3)	C12—C11—C10	120.7 (4)
N2—C2—S1	118.6 (3)	C12—C11—H11	119.6
C2—N2—N1	120.4 (3)	C10—C11—H11	119.6
C2—N2—H2	118 (3)	C13—C12—C11	120.0 (4)
N1—N2—H2	113 (3)	C13—C12—H12	120.0
C2—N3—C9	125.5 (3)	C11—C12—H12	120.0
C2—N3—H3	117 (3)	C12—C13—C14	120.0 (4)
C9—N3—H3	117 (3)	C12—C13—H13	120.0
C8—C3—C4	118.8 (3)	C14—C13—H13	120.0
C8—C3—C1	117.0 (3)	C15—C14—C13	119.7 (4)
C4—C3—C1	124.2 (3)	C15—C14—H14	120.2
C5—C4—C3	120.4 (3)	C13—C14—H14	120.2
C5—C4—H4	119.8	C10—C15—C14	121.1 (4)
C3—C4—H4	119.8	C10—C15—H15	119.5
C6—C5—C4	119.2 (3)	C14—C15—H15	119.5
C6—C5—H5	120.4	C9—C16—C17	111.8 (3)
C4—C5—H5	120.4	C9—C16—H16A	109.3
C5—C6—C7	121.5 (4)	C17—C16—H16A	109.3
C5—C6—Br1	119.0 (3)	C9—C16—H16B	109.3
C7—C6—Br1	119.5 (3)	C17—C16—H16B	109.3
C8—C7—C6	118.9 (3)	H16A—C16—H16B	107.9
C8—C7—H7	120.5	C16—C17—H17A	109.5
C6—C7—H7	120.5	C16—C17—H17B	109.5
C7—C8—C3	121.0 (3)	H17A—C17—H17B	109.5
C7—C8—H8	119.5	C16—C17—H17C	109.5
C3—C8—H8	119.5	H17A—C17—H17C	109.5
N3—C9—C16	109.8 (3)	H17B—C17—H17C	109.5
N3—C9—C10	110.8 (3)		
			
O1—C1—N1—N2	−13.7 (5)	C4—C3—C8—C7	−1.1 (6)
C3—C1—N1—N2	166.7 (3)	C1—C3—C8—C7	176.6 (3)
N3—C2—N2—N1	−26.7 (4)	C2—N3—C9—C16	−125.2 (3)
S1—C2—N2—N1	154.7 (2)	C2—N3—C9—C10	106.1 (4)
C1—N1—N2—C2	131.2 (3)	N3—C9—C10—C15	121.4 (4)
N2—C2—N3—C9	175.6 (3)	C16—C9—C10—C15	−4.2 (5)
S1—C2—N3—C9	−6.0 (4)	N3—C9—C10—C11	−58.7 (4)
O1—C1—C3—C8	−5.4 (5)	C16—C9—C10—C11	175.7 (3)
N1—C1—C3—C8	174.2 (3)	C15—C10—C11—C12	−0.8 (6)
O1—C1—C3—C4	172.1 (3)	C9—C10—C11—C12	179.3 (3)
N1—C1—C3—C4	−8.3 (5)	C10—C11—C12—C13	0.8 (6)
C8—C3—C4—C5	0.8 (5)	C11—C12—C13—C14	−0.1 (6)
C1—C3—C4—C5	−176.7 (3)	C12—C13—C14—C15	−0.5 (6)
C3—C4—C5—C6	0.2 (5)	C11—C10—C15—C14	0.2 (6)
C4—C5—C6—C7	−1.1 (6)	C9—C10—C15—C14	−180.0 (4)
C4—C5—C6—Br1	178.7 (3)	C13—C14—C15—C10	0.5 (6)
C5—C6—C7—C8	0.8 (6)	N3—C9—C16—C17	57.4 (4)
Br1—C6—C7—C8	−179.0 (3)	C10—C9—C16—C17	−176.5 (3)
C6—C7—C8—C3	0.3 (6)		

### Hydrogen-bond geometry (Å, °)

**Table:**

*D*—H···*A*	*D*—H	H···*A*	*D*···*A*	*D*—H···*A*
N1—H1···O1^i^	0.84 (4)	2.03 (4)	2.834 (4)	161 (4)

Symmetry codes: (i) −*x*, *y*+1/2, −*z*+1/2.
